# Blennorrhea Treated with Ichthyol

**Published:** 1897-06

**Authors:** 


### BLENNORRHEA TREATED WITH ICHTHYOL.

Dr. S. Werner, in *Monats. f. Prakt. Derm.* (1896, XXIII), gives the results of a series of investigations upon the use of ichthyol in the treatment of blennorrhea. He used the ichthyol in the form of one to two per cent. douches, and altogether treated one hundred and twenty patients with some fifteen hundred irrigations. Of all these patients, only eighty-eight could be kept under his control, however. The injections were made in such a manner as to dilate entirely the folds of the urethra, in order that the liquid might be brought into contact with every portion of the mucous membrane. At times the liquid passed into the bladder, and in such cases about 100 c.c. of the liquid were allowed to flow in, and then the patient was directed to urinate, the flow being occasionally checked by pressure at the orifice of the glans. This procedure was repeated once or twice. Altogether, it was found that 500 c.c. was a fair average for each sitting Cold solutions were preferable, since it is thought that in such solutions the disinfectant power is increased, and an astringent effect is also obtained.

Among the eighty-eight cases mentioned were six cases of acute vesical catarrh, and twenty in which the gonorrhea was confined to the pars anterior. The secretion was permanently free from gonococci within the first week of the treatment.

Six cases of acute vesical catarrh, and four cases of urethritis not due to gonorrhea, were also treated with ichthyol irrigations. In the latter cases, seven to nine applications suppressed the secretion and inflammation. In the former, most excellent results were obtained. After two irrigations the urinary tenesmus disappeared, the temperature declined, and the urine became clear; after eleven to fifteen douches, a permanent cure resulted.

The writer believes that also in vesical catarrhs accompanied with high fever, urinary tenesmus, and hematuria, the use of ichthyol is of great advantage.

Canova recommends a one or two per cent. solution of ichthyol as a urethral injection. He says it is painless and very efficient; in a few cases it has cured the disease in so short a time as six days.

---

HYDROGEN DIOXIDE IN THE TREATMENT OF EPISTAXIS.—Gillette (*New York Medical Journal*) recommends the use of hydrogen dioxide in cases of bleeding at the nose. He uses a teaspoonful or more in full strength, with any ordinary syringe. Relief is obtained immediately. In operations in the nasal cavity, when bleeding obscures the vision, inject hydrogen dioxide. Ask the patient to blow the nose, and the field is clear again.

